# BROCCOLI: Software for fast fMRI analysis on many-core CPUs and GPUs

**DOI:** 10.3389/fninf.2014.00024

**Published:** 2014-03-14

**Authors:** Anders Eklund, Paul Dufort, Mattias Villani, Stephen LaConte

**Affiliations:** ^1^Virginia Tech Carilion Research Institute, Virginia TechRoanoke, VA, USA; ^2^Department of Medical Imaging, University of TorontoToronto, ON, Canada; ^3^Division of Statistics, Department of Computer and Information Science, Linköping UniversityLinköping, Sweden; ^4^School of Biomedical Engineering and Sciences, Virginia Tech-Wake Forest UniversityBlacksburg, VA, USA

**Keywords:** Neuroimaging, fMRI, Spatial normalization, GPU, CUDA, OpenCL, Image registration, Permutation test

## Abstract

Analysis of functional magnetic resonance imaging (fMRI) data is becoming ever more computationally demanding as temporal and spatial resolutions improve, and large, publicly available data sets proliferate. Moreover, methodological improvements in the neuroimaging pipeline, such as non-linear spatial normalization, non-parametric permutation tests and Bayesian Markov Chain Monte Carlo approaches, can dramatically increase the computational burden. Despite these challenges, there do not yet exist any fMRI software packages which leverage inexpensive and powerful graphics processing units (GPUs) to perform these analyses. Here, we therefore present BROCCOLI, a free software package written in OpenCL (Open Computing Language) that can be used for parallel analysis of fMRI data on a large variety of hardware configurations. BROCCOLI has, for example, been tested with an Intel CPU, an Nvidia GPU, and an AMD GPU. These tests show that parallel processing of fMRI data can lead to significantly faster analysis pipelines. This speedup can be achieved on relatively standard hardware, but further, dramatic speed improvements require only a modest investment in GPU hardware. BROCCOLI (running on a GPU) can perform non-linear spatial normalization to a 1 mm^3^ brain template in 4–6 s, and run a second level permutation test with 10,000 permutations in about a minute. These non-parametric tests are generally more robust than their parametric counterparts, and can also enable more sophisticated analyses by estimating complicated null distributions. Additionally, BROCCOLI includes support for Bayesian first-level fMRI analysis using a Gibbs sampler. The new software is freely available under GNU GPL3 and can be downloaded from github (https://github.com/wanderine/BROCCOLI/).

## 1. Introduction

Functional magnetic resonance imaging (fMRI) has become the de facto standard methodology in contemporary efforts to image the functioning of the human brain in both health and disease. Nonetheless, fMRI-based research arguably lags behind in its adoption of recent advances in computer hardware, despite several recent trends that have underlined the need for greater computational resources. First, the temporal and the spatial resolution of fMRI data continues to improve with stronger magnetic fields and more advanced scanning protocols (Moeller et al., 2010; Feinberg and Yacoub, 2012), leading to the production of significantly larger datasets. Second, fMRI studies are trending toward larger numbers of subjects to increase their statistical power (Eklund et al., 2012a; Thyreau et al., 2012; Button et al., 2013) sometimes aided by a proliferation of data sharing initiatives (Biswal et al., 2010; Poldrack et al., 2013)^1^^,^^2^ that provide open access to large amounts of data. The human connectome project (van Essen et al., 2013)^3^, for example, shares high resolution data from a large number of subjects (the goal is 1200), and a single resting state scan results in a dataset of the size 104 × 90 × 72 × 1200. Third, non-parametric methods based on permutation and Bayesian Markov Chain Monte Carlo (MCMC) methods are more frequently being used to improve neuroimaging statistics (da Silva, 2011; Eklund et al., 2012a, 2013b), but suffer from long processing times compared to conventional parametric methods. Some progress toward parallelization has been made in each of the three major packages commonly used in fMRI-based research (SPM, FSL, and AFNI). For example, AFNI has direct support for running some functions in parallel on several CPU cores, using the open multi-processing (OpenMP) library; FSL can take advantage of several computers or CPU cores, by installing packages like Condor or GridEngine, and has recently added graphics processing unit (GPU) support for MCMC based diffusion tensor analysis (Hernandez et al., 2013); and Huang et al. (2011) recently proposed to accelerate image registration in SPM by using a GPU. Moreover, a number of prominent projects are underway to enable big data approaches to functional neuroimaging at large supercomputing centers [e.g., (Lavoie-Courchesne et al., 2012)]. At this stage, however, these approaches still require a significant investment of time and effort by expert technical staff, and thus remain inaccessible to the majority of investigators. Thus, despite efforts by existing analysis packages, we feel that the community could benefit from a more comprehensive focus on parallel computation. Further, being relatively new, GPUs offer some unique challenges as well as promising potential benefits.

Since the introduction of the CUDA programming language in 2007, general purpose computing on graphics processing units (GPGPU) (Owens et al., 2007) has gained prominence in a wide range of scientific fields, including medical imaging (Shams et al., 2010; Pratx and Xing, 2011; Eklund et al., 2013a) and neuroscience (Jeong et al., 2010; Pezoa et al., 2012; Ben-Shalom et al., 2013; Hoang et al., 2013; Yamazaki and Igarashi, 2013). The main reasons are that GPUs are inexpensive, power efficient and able to run several thousand threads in parallel, commonly providing a performance boost of 1–2 orders of magnitude for a small investment (see Table 1). Nonetheless, GPGPU is still uncommon in the neuroimaging field, where medical imaging and neuroscience intersect. Here, we therefore present BROCCOLI, a free software for parallel analysis of fMRI data on many-core CPUs and GPUs. BROCCOLI contains a large number of additions and improvements over our previous work (Eklund et al., 2010, 2011a; Forsberg et al., 2011; Eklund et al., 2012b). Some examples are Bayesian fMRI analysis using MCMC, first level statistical analysis using the Cochrane-Orcutt procedure (Cochrane and Orcutt, 1949), linear and non-linear registration for an arbitrary number of scales and support for *F*-tests as well as a larger number of regressors. While our previous implementations used CUDA, the most popular programming language for GPGPU, BROCCOLI is instead written in the open computing language (OpenCL) [see e.g., Munshi et al. (2011)]. This makes it possible to run BROCCOLI on many types of hardware, including CPUs, Nvidia GPUs, AMD GPUs, field programmable gate arrays (FPGAs), digital signal processors (DSPs) and other accelerators (e.g., the Intel Xeon Phi). As neuroimaging researchers use a wide range of operating systems (Hanke and Halchenko, 2011), it is also important that BROCCOLI can run efficiently regardless of the platform. One way to achieve this is to develop BROCCOLI for a specific platform (e.g., Windows), and then simply run BROCCOLI through a virtual machine for other platforms (e.g., Linux). However, direct access to GPU hardware through a virtual machine can currently be problematic, and was therefore not an option for our software. Instead, we have developed BROCCOLI using a combination of the platform-independent languages OpenCL and C++, and have made the source code freely available so that it can be compiled on any desired operating system supporting these widely deployed standards. In addition, as an added convenience, we have provided pre-compiled libraries for the Linux and Windows operating systems that can be linked to projects developed on either platform. A wrapper for Matlab is currently available, a Python wrapper is being developed and future plans include wrappers for bash and R. In addition to the improvements described above, BROCCOLI has also been extensively tested and compared to SPM, FSL, and AFNI by using a large number of freely available fMRI datasets. BROCCOLI is available as free software under GNU GPL3 and can be downloaded from github^4^.

**Table 1:** **Hardware configuration and performance measures of the computer used for testing the different software packages**.

**Device**	**Processor cores**	**Memory (GB)**	**Single precision (GFLOPS)**	**Double precision (GFLOPS)**	**Memory bandwidth (GB/s)**	**Price (USD)**
Intel Core i7-3770K	4 (8 with hyper threading)	16	1 core: 56, 4 cores: 224	1 core: 28, 4 cores: 112	26	330
Nvidia GTX 680	1536	4	3090	129	192	500
AMD Radeon 7970	2048	3	3790	947	264	500

A Linux operating system was used (CentOS 6.4 64 bit) with an OCZ 128 GB SSD hard drive. The theoretical performance for single (32 bit floats) and double (64 bit floats) precision is given as giga floating point operations per second (GFLOPS). Prices are from newegg.com and should be seen as approximate.

## 2. Methods and implementation

The typical analysis pipeline for fMRI data is compromised of image registration, image segmentation, slice timing correction, smoothing, and statistical analyses. The methods used for these different processing steps in BROCCOLI are described in this section, and implementation details are given at the end of the section.

### 2.1. Image registration

Image registration for fMRI is used to align an anatomical T1 volume to a brain template (e.g., MNI or Talairach), to align an fMRI volume to the anatomical T1 volume, and to perform motion correction. The registration between the anatomical space and a standard brain space, often called spatial normalization, can be performed using a linear transformation model (e.g., affine or rigid) or by using a non-linear approach, which is much more computationally demanding. In a comparison of non-linear deformation algorithms for human brain MRI registration (Klein et al., 2009), the DARTEL algorithm in SPM took an average of 71 min to register a single T1 volume to the MNI template (1 mm^3^ resolution) and the FNIRT algorithm in FSL used an average of 29 min. The AFNI software did not until recently have support for non-linear registration, but can now be achieved through the function 3dQwarp. Based on our benchmarking, non-linear registration with 3dQwarp takes about 36 min with a single-threaded version of AFNI, and 13 min using the multi-threaded OpenMP version (for a CPU running 8 threads). Thus, depending on the algorithm, normalization for a study involving 30 subjects can take 5–35.5 h. Moreover, to obtain satisfactory results, it may be necessary to run the registration algorithm with a number of different settings. For these reasons, affine registration to a standard brain space is sometimes performed instead of a non-linear one, even though the non-linear approach can yield a better registration. Another time saving approach is to perform spatial normalization to a brain template of lower resolution, e.g., 2 mm^3^ voxels, but this solution is less appealing, since spatial resolution is sacrificed. Due to the computational challenges of image registration, GPU acceleration of such algorithms is very popular with some 60 publications since 1998 (Shams et al., 2010; Fluck et al., 2011; Pratx and Xing, 2011; Eklund et al., 2013a). GPUs can thus easily be used in the neuroimaging field, to for example enable more widespread use of demanding non-linear registration algorithms.

#### 2.1.1. Linear image registration

BROCCOLI uses a single registration algorithm to perform the three described registrations (T1-to-MNI,fMRI-to-T1, and motion correction). Here we summarize the algorithm, which has been previously described (Eklund et al., 2010). The main idea of the algorithm is to use the optical flow equation (Horn and Schunck, 1981)

(1) $$\nabla I^{T}v=\Delta I,$$

where ∇*I* is the gradient of the volume, ***v*** is a motion vector that describes the difference between the volumes and Δ*I* is the intensity difference between the two volumes. The aperture problem, however, prevents us from solving this equation directly, as there are three unknown variables (the motion in x, y, and z), but only one equation. Instead of solving the equation for each voxel separately, one can minimize the expression over the entire volume. The total squared error can be written as

(2) $$\epsilon^{2}=\sum_{i}{\left(\nabla I{\left(x_{i}\right)}^{T}v(x_{i})-\Delta I(x_{i})\right)}^{2},$$

where ***x***_*i*_ denotes the position of voxel *i*. A linear model of the motion field can be used to represent a motion vector in each voxel. The motion field ***v***(***x***) for affine transformations in 3D can be modelled with a 12-dimensional parameter vector, ***p*** = [*p*_1_, *p*_2_, *p*_3_, *p*_4_, *p*_5_, *p*_6_, *p*_7_, *p*_8_, *p*_9_, *p*_10_, *p*_11_, *p*_12_]^*T*^, and a base matrix ***B***(***x***) according to (Hemmendorff et al., 2002)

(3) $$v(x)=\left[\begin{array}{c} p_{1}\\ p_{2}\\ p_{3} \end{array}\right]+\left[\begin{array}{lll} p_{4} & p_{5} & p_{6}\\ p_{7} & p_{8} & p_{9}\\ p_{10} & p_{11} & p_{12} \end{array}\right]\left[\begin{array}{c} x\\ y\\ z \end{array}\right]$$

$$=\underset{B}{\underset{︸}{\left[\begin{array}{llllllllllll} 1 & 0 & 0 & x & y & z & 0 & 0 & 0 & 0 & 0 & 0\\ 0 & 1 & 0 & 0 & 0 & 0 & x & y & z & 0 & 0 & 0\\ 0 & 0 & 1 & 0 & 0 & 0 & 0 & 0 & 0 & x & y & z \end{array}\right]}}p.$$

The first three parameters are the translations and the last nine parameters form a transformation matrix (if an identity matrix is added, as the parameter vector ***p*** used here only describes the difference between the two volumes). The variables *x*, *y*, and *z* are the coordinates of voxel *x*. By using the model of the motion field, ***v***(***x***) = ***B***(***x***) ***p***, the error measure can be written as

(4) $$\epsilon^{2}=\sum_{i}{\left(\nabla I{\left(x_{i}\right)}^{T}B(x_{i})\;p-\Delta I(x_{i})\right)}^{2}.$$

The derivative of this expression, with respect to the parameter vector, is given by

(5) $$\frac{\partial \epsilon^{2}}{\partial p}=2\sum_{i}B_{i}^{T}\nabla I_{i}\left(\nabla I_{i}^{T}B_{i}\;p-\Delta I_{i}\right),$$

and setting the derivative to zero yields the following linear equation system

(6) $$\underset{A}{\underset{︸}{\sum_{i}B_{i}^{T}\nabla I_{i}\nabla I_{i}^{T}B_{i}}}p=\underset{h}{\underset{︸}{\sum_{i}B_{i}^{T}\nabla I_{i}\Delta I_{i}}},$$

where ***A*** is a matrix of size 12 × 12 and ***h*** is a vector of size 12 × 1. The best parameter vector can finally be calculated as

(7) $$p=A^{-1}h.$$

The system of linear equations is easy to solve, while the computationally demanding part is to sum over all voxels. *L*_2_ norm minimization makes it possible to calculate the parameters that give the best solution. The solution can then be improved by iterating the algorithm and accumulating the parameter vector (to avoid repeated interpolation). The most common approach is otherwise to maximize a similarity measure by searching for the best parameters, using some optimization algorithm. To handle large differences between two volumes, it is common to start the registration on a coarse scale and then improve the registration by moving to finer scales. BROCCOLI uses three to four scales for the registration between T1 and MNI and between fMRI and T1; the difference between each scale is a factor two in each dimension.

The estimated affine transformation parameters can be restricted to a rigid transformation (i.e., translations and rotations only), and is accomplished in BROCCOLI by applying a singular value decomposition (SVD) to the transformation matrix and then forcing the singular values to be one. Rigid registration is used for fMRI-T1 registration and for motion correction, while affine registration (12 parameters) is used for the T1-MNI registration. For the motion correction procedure, the rotation angles θ_1_, θ_2_, θ_3_ are extracted from the estimated rotation matrix for each time point using the following formulas (Shoemake, 1994; Day, 2012)

(8) $$\begin{array}{l} \theta_{1}=atan2(p_{9},p_{12}),\\ c_{2}=\sqrt{p_{4}\cdot p_{4}+p_{5}\cdot p_{5}},\\ \theta_{2}=atan2(-p_{6},c_{2}),\\ s_{1}=sin(\theta_{1}),\\ c_{1}=cos(\theta_{1}),\\ \theta_{3}=atan2(s_{1}\cdot p_{10}-c_{1}\cdot p_{7},c_{1}\cdot p_{8}-s_{1}\cdot p_{11}), \end{array}$$

where *atan*2(*a*, *b*) is the four quadrant arctangent of *a* and *b*. The main reasons for extracting the rotation angles are to use them as nuisance regressors in the statistical analysis and to present them to the user.

#### 2.1.2. Non-intensity based image registration

**Figure 1:**
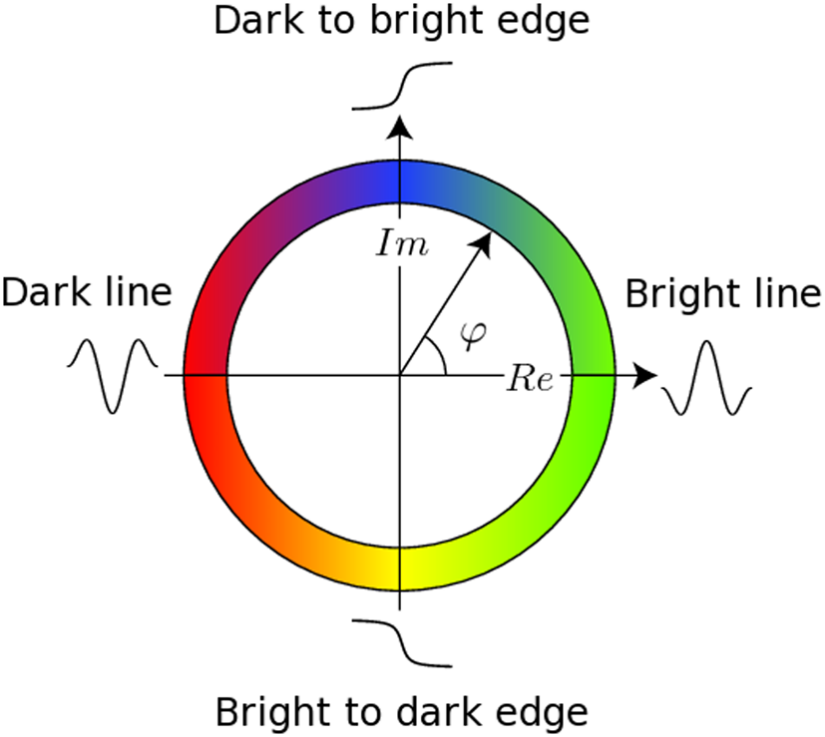
**This figure presents the main concept of local phase φ from quadrature filter responses.** A quadrature filter is complex valued in the spatial domain; the real part is a line detector and the imaginary part is an edge detector. If the filter response only contains a real valued component, it means that the filter detected a line. If the filter response only contains an imaginary valued component, it means that the filter detected an edge. It is important to combine the local phase with the magnitude of the complex valued filter response, as the local phase does not have any meaning for a low magnitude.

The registration algorithm used in BROCCOLI is not based on the image intensity directly, e.g., the image gradient as described above. Instead, the algorithm is based on matching edges to edges and lines to lines, by using the concept of local phase from quadrature filter responses (Granlund and Knutsson, 1995; Knutsson and Andersson, 2003). A quadrature filter is complex valued in the spatial domain; the real part is a line detector and the imaginary part is an edge detector. The local phase is the relationship between the real and imaginary filter responses and describes the type of local structure (e.g., a line or an edge), while the magnitude can be seen as a certainty measure of how likely it is that the filter detected a structure. The local phase concept is illustrated in Figure 1. The quadrature filters need to be created using filter optimization techniques, which simultaneously consider properties in the spatial domain and the frequency domain (Granlund and Knutsson, 1995; Knutsson et al., 1999). In the presented equations, the image gradient ∇*I* is replaced with a phase gradient ∇φ and the image difference Δ*I* is replaced with a phase difference Δφ. The phase difference can be calculated as

(9) $$\Delta \varphi =arg\left(q_{1}\cdot q_{2}^{*}\right),$$

where *q*_1_ and *q*_2_ are the complex valued quadrature filter responses for the two volumes and ∗ denotes complex conjugation. A nice property of the local phase is that it is invariant to the image intensity (all edges are for example interpreted equally, regardless if the image intensity changes from 0 to 1 or from 10 to 11), making it easier to register volumes from different modalities or volumes with different or varying contrast. Phase based optical flow was introduced in the field of computer vision (Fleet and Jepson, 1990) and eventually propagated to the medical imaging domain (Hemmendorff et al., 2002; Knutsson and Andersson, 2005; Mellor and Brady, 2005). While phase based image registration can in some cases be more robust against intensity differences (Hemmendorff et al., 2002; Mellor and Brady, 2005; Eklund et al., 2011b), a drawback is that it requires filtering with a number of (non-separable) filters in each iteration, which is computationally demanding. Fortunately, GPUs are perfectly suited for parallel operations like filtering (Eklund and Dufort, 2014).

#### 2.1.3. Non-linear image registration

As previously mentioned, non-linear methods can lead to a significantly better registration between a subject specific anatomical volume and a brain template. BROCCOLI uses the Morphon (Knutsson and Andersson, 2005; Forsberg et al., 2011; Forsberg, 2013) to perform non-linear registration. The Morphon is also based on phase based optical flow, and the two most important parts of the Morphon are, therefore, the same as for the linear registration algorithm; to apply a number of quadrature filters and to calculate phase differences. The main differences are that the linear algorithm uses three quadrature filters (oriented along x, y, and z) and solves one equation system for the entire volume, while the Morphon uses six quadrature filters (evenly distributed on the half sphere of an icosahedron) and solves as many equation systems as there are voxels. The error being minimized in each voxel can be written as

(10) $$\epsilon^{2}=\sum_{k=1}^{N}{\left(c_{k}T(\Delta \varphi_{k}{\hat{n}}_{k}-d)\right)}^{2},$$

where Δφ_*k*_ is the phase difference between the two volumes for quadrature filter *k*, *c*_*k*_ is a certainty estimate for filter *k*, ${\hat{n}}_{k}$ is the orientation vector for filter *k*, *N* is the number of quadrature filters, ***d*** is the displacement vector to be optimized and ***T*** is a local structure tensor (Knutsson, 1989; Granlund and Knutsson, 1995; Knutsson et al., 2011). A local structure tensor in image processing is analogous to a diffusion tensor in diffusion tensor imaging (DTI); it represents the magnitude and orientation of the signal in each neighborhood. The tensor can be calculated from the six complex valued quadrature filter responses as (Granlund and Knutsson, 1995)

(11) $$T=\sum_{k=1}^{N}|q_{k}|(\frac{5}{4}{\hat{n}}_{k}{\hat{n}}_{k}^{T}-\frac{1}{4}I),$$

where ***I*** is an identity tensor. The purpose of using the tensor in the error measure is to reinforce displacement estimates along the local predominant orientations (i.e., displacements perpendicular to edges and lines). Using an *L*_2_-norm, the best displacement vector can be calculated for each voxel directly, by once again solving a linear system (of size 3 x 3), i.e.,

(12) $$d={\left(\sum_{k=1}^{N}c_{k}^{2}T^{T}T\right)}^{-1}\sum_{k=1}^{N}c_{k}^{2}\Delta \varphi_{k}T^{T}T{\hat{n}}_{k}.$$

The estimated displacement field is regularized by applying Gaussian smoothing separately to each motion component (x, y, z) before it is used to warp the T1 volume. Just as for the linear registration, the displacement field is accumulated in each iteration to avoid repeated interpolation. An affine registration (12 parameters) is first estimated between the T1 volume and the MNI template before estimation of the non-linear displacement field.

### 2.2. Image segmentation

SPM has several functions for segmenting brain volumes. FSL provides BET (brain extraction tool) and FAST (FMRIB's automated segmentation tool) while AFNI provides the function 3dSkullStrip. BROCCOLI performs skullstripping by first registering the T1 volume to MNI space, using an MNI template with skull, then applies an inverse transform to the MNI brain mask and finally performs a multiplication between the transformed mask and the original T1 volume to obtain a skullstripped version of the T1 volume. The skullstripped T1 volume is then aligned to an MNI template without skull, to improve the alignment, and the MNI brain mask is again inversely transformed (using the new registration parameters) and multiplied with the original T1 volume, to obtain a better skullstrip. The fMRI data is segmented by first applying 4 mm 3D Gaussian smoothing to one of the fMRI volumes and then using a threshold that is 90% of the mean value.

### 2.3. Slice timing correction

Slice timing correction is normally applied to fMRI data (Sladky et al., 2011), as the slices in each volume are collected at slightly different time points. BROCCOLI sets the middle slice as the reference and then applies cubic interpolation in time to correct for the temporal difference between the slices.

### 2.4. Smoothing

fMRI data is frequently spatially smoothed. The non-linear registration algorithm also uses Gaussian smoothing, for example to reguralize the tensor components and the resulting displacement field in each iteration. BROCCOLI utilizes a simple form of normalized convolution (Knutsson and Westin, 1993), called normalized averaging, to avoid problems with voxels close to the edge of the brain being influenced by voxels outside the brain. The normalized filter response *nfr* is calculated as

(13) $$nfr=\frac{(v\cdot c)*f}{c*f},$$

where *f* is the filter, *v* is one fMRI volume, *c* is a certainty measure, ∗ denotes convolution and · denotes pointwise multiplication. The certainty is simply the fMRI brain mask, such that the certainty is one inside the brain and zero outside. If a gray matter segmentation is available, the same approach can be used to prevent similar problems with smoothing that includes values from other types of brain matter (by setting the certainty to one for gray voxels and zero for all other voxels).

### 2.5. Statistical analysis

The statistical analysis is the core of all fMRI software packages. The use of GPUs for statistical computations is a relatively new concept (Suchard et al., 2010; Guo, 2012) and can for example be used to speedup demanding Markov Chain Monte Carlo (MCMC) simulations (Lee et al., 2010). We believe that GPUs (or at least the computational capacity they confer) are a necessary component for incorporation of developments in the field of statistics to the field of neuroimaging, especially for high resolution fMRI data (Feinberg and Yacoub, 2012). By using GPUs, computationally demanding non-parametric tests can be used instead of parametric ones (Nichols and Holmes, 2002; Eklund et al., 2011a) and MCMC based methods [e.g., (Woolrich et al., 2004)] also become feasible (da Silva, 2011).

The SPM, FSL, and AFNI software packages are all mainly based on the general linear model (GLM) for first (subject) and second level (group) analyses, as proposed by Friston et al. (1994). The GLM can be written in matrix form as

(14) y=Xβ+ϵ,

where ***y*** are the observations for one voxel, **β** are the parameters to estimate, ***X*** is the design matrix (model) containing all the regressors and **ϵ** are the errors that cannot be explained by the model. As the GLM is applied to each voxel independently, it is perfectly suited for parallel implementations. By minimizing the squared error ||**ϵ**||^2^, it can be shown that the best parameters (for independent errors) are given by

(15) $$\hat{\beta }={\left(X^{T}X\right)}^{-1}X^{T}y.$$

A useful property of this expression is that the term (***X***^*T*^ ***X***)^−1^ ***X***^*T*^ is the same for all voxels and can, thus, be precalculated. A *t*-test value can easily be calculated from the estimated weights as

(16) $$t=\frac{c^{T}\hat{\beta }-u}{\sqrt{\text{var}\left(\hat{\epsilon }\right)\;c^{\text{T}}{\left(X^{\text{T}}X\right)}^{\text{-1}}c}},$$

where ***c*** is a contrast vector, $\hat{\epsilon }$ is the residual of the GLM and *u* is a scalar for the null hypothesis $c^{T}\hat{\beta }=u$. An *F*-test value can in a similar manner be calculated as

(17) $$F=\frac{{\left(C\hat{\beta }-u\right)}^{T}{\left(\text{var}\left(\hat{\epsilon }\right)\;C{\left(X^{\text{T}}X\right)}^{\text{-1}}C^{\text{T}}\right)}^{-1}\left(C\hat{\beta }-u\right)}{N},$$

where ***C*** is a contrast matrix and *N* is the number of contrasts.

#### 2.5.1. First level analysis

The first level fMRI analysis starts with slice timing correction and motion correction. The estimated motion parameters (translations and rotations) are included in BROCCOLI by default as additional regressors in the GLM design matrix, to further reduce effects of head motion (Johnstone et al., 2006). Gaussian smoothing is applied to each fMRI volume and the GLM is finally applied to the smoothed volumes. In addition to motion regressors and regressors for the experimental design, the design matrix in BROCCOLI also contains regressors to remove the mean and trends that are linear, quadratic or cubic. The effect of using these additional regressors is similar to a highpass filtering. The GLM errors are for first level fMRI analysis often modelled as an auto regressive (AR) process,

(18) $$\epsilon_{t}=\sum_{i=1}^{p}\rho_{i}\epsilon_{t-i}+w_{t},$$

where *p* is the order of the AR process, ρ_*i*_ are the AR parameters and *w* is white noise with variance σ^2^. A Cochrane-Orcutt procedure (Cochrane and Orcutt, 1949) is used in BROCCOLI to estimate the beta weights for autocorrelated errors. The GLM weights **β** are first estimated using ordinary least squares (equation 15) and then a voxel-wise AR model of the fourth order is used to model the residuals (Worsley et al., 2002). The AR parameters are estimated by solving the Yule-Walker equations independently for each voxel. Each volume of AR estimates is spatially smoothed with a 7 mm Gaussian filter to further improve the estimates (Woolrich et al., 2001; Worsley et al., 2002; Gautama and Hulle, 2004), before the actual whitening is applied to the smoothed fMRI data and the regressors in the design matrix (such that each voxel gets its own specific design matrix). The components of the whitened data $\tilde{y}$ and the whitened regressors $\tilde{X}$ are thus calculated as

(19) $${\tilde{y}}_{t}=y_{t}-\sum_{i=1}^{4}\rho_{i}y_{t-i},$$

(20) $${\tilde{X}}_{t,\;r}=X_{t,\;r}-\sum_{i=1}^{4}\rho_{i}X_{t-i,\;r},$$

where ρ_*i*_ are the spatially smoothed AR estimates, *r* denotes regressor and *t* denotes time point. The whitened data $\tilde{y}$ and the whitened regressors $\tilde{X}$ are then used to estimate new beta weights, according to

(21) $$\tilde{\beta }={\left({\tilde{X}}^{T}\tilde{X}\right)}^{-1}{\tilde{X}}^{T}\tilde{y}.$$

As a last step, the AR parameters are re-estimated using residuals calculated with the new weights $\tilde{\beta }$, the original data ***y*** and the original regressors ***X***. The Cochrane-Orcutt procedure is repeated three times to obtain good estimates of the GLM weights and the AR parameters. Finally, the statistical maps are calculated using the variance of the uncorrelated residuals $\tilde{\epsilon }$, obtained as

(22) $$\tilde{\epsilon }=\tilde{y}-\tilde{X}\tilde{\beta }.$$

FSL uses a similar iterative approach to estimate a voxel-wise prewhitening matrix (Woolrich et al., 2001), with the exception that the spatial smoothing is done separately for different tissue types. The voxel-specific noise model used in BROCCOLI has been shown to yield more valid results than those obtained from SPM (Eklund et al., 2012a), which uses a global AR(1) model. After the first level statistical analysis, the results (e.g., beta weights) are transformed to MNI space, by combining the estimated registration parameters for T1-to-MNI and fMRI-to-T1 transformations and the estimated displacement field from the non-linear registration.

#### 2.5.2. Second level analysis

The second level analysis in fMRI is straightforward compared to the first level analysis, once all the first level results are in a common brain space. A group-wise *t*-test or *F*-test can easily be performed by using the same functions as for the first level GLM. BROCCOLI currently only supports conventional *t*-tests and *F*-tests for second level analysis, but we plan to also include other types of analyses (e.g., where the variance of the beta estimates are used as weights) in future releases.

#### 2.5.3. Frequentist inference

In contrast to other software packages for fMRI analysis, BROCCOLI is not based on parametric statistics. All *p*-values are instead calculated through non-parametric permutation tests (Dwass, 1957; Nichols and Holmes, 2002), both for first level and second level analyses. The main motivation is that parametric statistics require several assumptions to be met for the results to be valid. In fMRI it is also necessary to correct for a large number of tests, due to the high spatial resolution. The multiple testing makes the parametric assumptions much more critical, as one has to move far along the tail of the null distribution. The SPM software relies on Gaussian random field theory (GRFT) to correct for the multiple testing (Worsley et al., 1992), while FSL mainly works with GRFT and non-parametric permutation tests (for group analyses only). AFNI instead uses the false discovery rate (FDR) (Genovese et al., 2002) and a cluster simulation tool. A permutation test solves the problem of multiple testing in a very simple way. In each permutation, only the largest value of the statistical map (e.g., the maximum *t*-test value, the maximum *F*-test value, the size or mass of the largest cluster etc.) is saved to form the null distribution of the maximum test statistics. Corrected *p*-values are finally calculated as the proportion of values in the estimated null distribution that are larger than or equal to the test value for the current voxel or cluster. A threshold for a certain significance level α, corrected for multiple testing, can be calculated by first sorting the estimated null distribution values, and then simply using the value that is larger than (100 − α) % of the values. The main problem is that a large number of permutations, normally 1000–10,000, are required to obtain a good estimate of the null distribution. Since a full statistical analysis needs to be performed in each permutation, the total processing time can be several hours or days for a single test, using conventional multi-core CPU implementations. This is the main reason why permutation tests are not standard procedure in the neuroimaging field.

For first level analysis in BROCCOLI, detrending and whitening [using a voxel-wise AR(4) model as previously described] is applied to the motion corrected data and new fMRI data is then generated in each permutation by using an inverse whitening transform with randomly permuted whitened data. The smoothing has to be applied in each permutation, as the smoothing alters the autocorrelation structure of the fMRI data. Permutation testing for the second level analysis is much easier, as no whitening or smoothing is required. See our previous work for further information on the non-parametric analysis (Eklund et al., 2011a, 2012a).

#### 2.5.4. Bayesian inference

The GLM model previously described can alternatively be analyzed using Bayesian methods. A Bayesian analysis begins with a prior distribution *p*(β, σ^2^, ρ) over the model parameters and subsequently updates the prior with the observed data. The result is the posterior distribution *p*(β, σ^2^, ρ|***X***, ***y***), which encapsulates all information about the unknown parameters conditional on the observed data. In fMRI, the brain activity can be visualized as a heat map of Pr(β_*i*_ > 0|***X***, ***y***), commonly known as a posterior probability map (PPM) (Friston et al., 2002). The joint posterior *p*(β, σ^2^, ρ|***X***, ***y***) is often not tractable in analytical form, but can be approximated by different approaches. The most common approach in the fMRI field is to use approximation techniques like variational Bayes, where the posterior is factorized into several independent factors to obtain an analytical expression (Penny et al., 2003). A less common approach is to use techniques based on Markov Chain Monte Carlo (MCMC) simulation. MCMC produces a sample from the posterior, and the probability of activity Pr(β_*i*_ > 0|***X***, ***y***) can be approximated by the proportion of simulated β_*i*_ being larger than zero. The PPM for any contrast is also directly available from the posterior simulations. Note that since simulations are done using the joint posterior, PPMs are not conditional on point estimates of σ^2^ and ρ, leading to more accurate inferences regarding brain acitivity.

BROCCOLI uses a specific MCMC algorithm, the Gibbs sampler, to generate draws from the posterior by iteratively simulating from two full conditional posteriors. First, the autocorrelation parameters ρ are updated by simulation from ρ|β, σ^2^, ***y***, ***X*** as a (multivariate) Gaussian distribution. Second, the variance σ^2^ is updated by simulation from σ^2^|ρ, ***y***, ***X*** as an inverse Gamma distribution and the GLM weights β are finally updated by simulation from β|σ^2^, ρ, ***y***, ***X*** as a (multivariate) Gaussian distribution. These conditional distributions are obtained when the priors for β|σ^2^ and ρ are Gaussian and the prior on σ^2^ is inverse Gamma. The exact details of each updating step can be found in most Bayesian textbooks, see e.g., Murphy (2012). Note that each updating step conditions on the most recently simulated value for the conditioning parameters. While MCMC methods can theoretically be used to approximate any posterior, a common problem is the significantly longer processing time compared to techniques like variational Bayes. BROCCOLI runs a large number of MCMC chains in parallel to reduce the processing time.

### 2.6. Implementation

We will here describe the implementation of BROCCOLI for the different algorithms. Readers are referred elsewhere for introductions to GPU programming (Kirk and Hwu, 2010; Munshi et al., 2011; Sanders and Kandrot, 2011). Most of the OpenCL code uses single precision to achieve maximum performance, while some host code uses double precision (to for example obtain the optimal affine registration parameter vector). The open source library Eigen^5^^,^^6^ is used in BROCCOLI to perform matrix calculations on the host.^7^

#### 2.6.1. Image registration

The described linear and non-linear registration algorithms are easy to run in parallel. The filtering operation applied in each iteration is the most demanding part, especially since quadrature filters are non-separable, and has therefore been carefully optimized. Filtering can be performed as a multiplication in the frequency domain, after the application of a fast Fourier transform (FFT) to the signal and the filter, or as a convolution in the spatial domain. BROCCOLI uses the convolution approach, for three reasons. First, the FFT approach requires an FFT library while the convolution approach can rather easily be implemented manually. The CUDA programming language provides the CUFFT library, and a similar OpenCL library called clFFT has recently appeared. However, clFFT is in our opinion not yet as mature as CUFFT. The user, for example, has to compile the whole project to obtain a library file. Second, a convolution approach often provides high performance over a wide range of data sizes, while an FFT normally performs best for data sizes being a power of 2. Third, the convolution approach is less memory demanding as the FFT approach requires that the filters are stored as the size of the signal for an elementwise multiplication.

**Figure 2:**
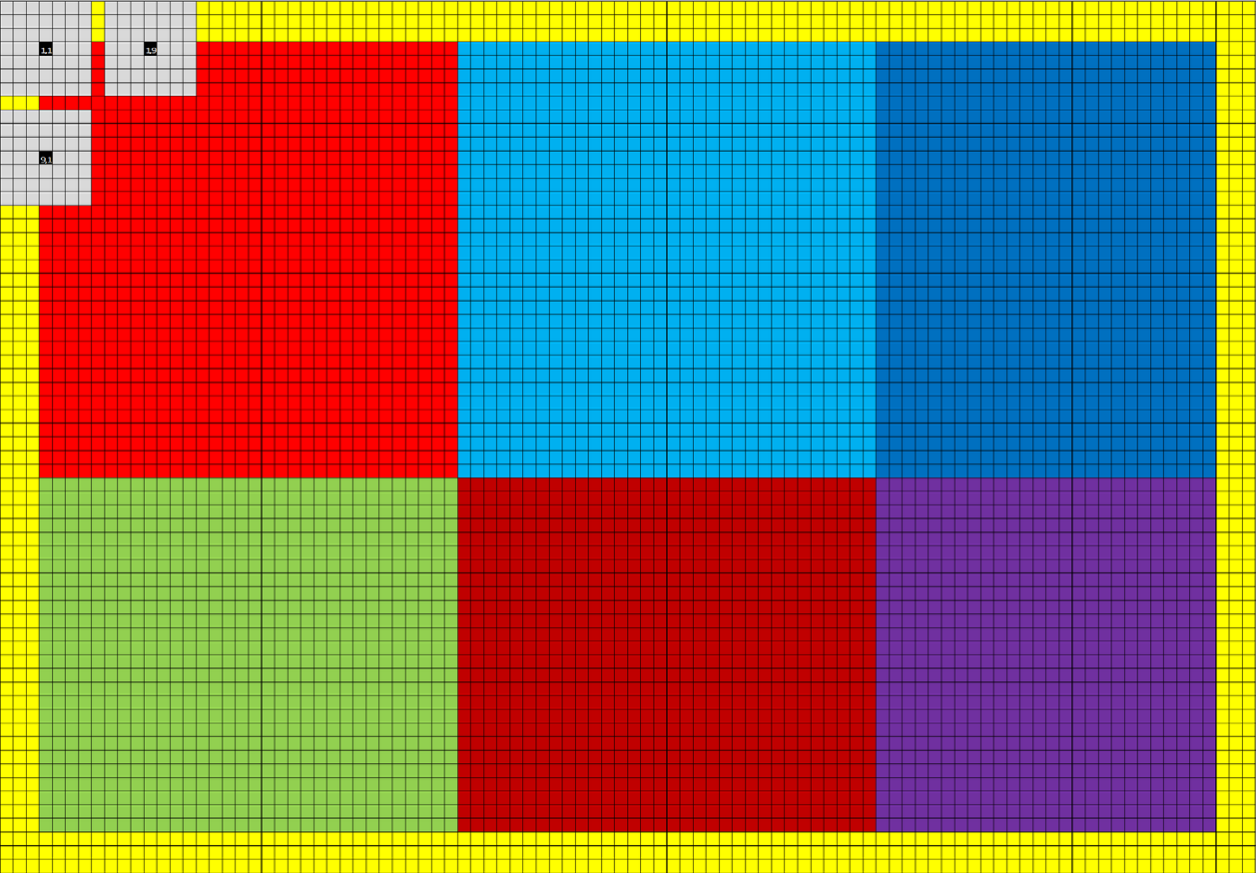
**The grid represents 96 × 64 pixels in local memory (each square is one pixel).** As 32 x 32 threads are used per thread block, each thread needs to read 6 values from global memory into local memory [(96 × 64)/(32 × 32) = 6]. A yellow halo needs to be loaded into local memory to be able to calculate all the filter responses. In this case 90 × 58 valid filter responses are calculated, making it possible to apply at most a filter of size 7 × 7. The 90 × 58 filter responses are calculated as 6 runs, the first 2 consisting of 32 × 32 pixels (marked light red and light blue). The 1024 filter responses (32 × 32) are calculated in parallel, and the gray squares represent three filter responses being calculated. Note that neighboring filter responses are calculated using mainly the same pixels. Three additional filter responses are calculated in blocks of 32 × 26 or 26 × 32 pixels (marked green, dark blue and dark red). Finally, a block of 26 × 26 pixels is processed (marked purple). The halo can easily be changed to handle larger filters.

Convolution is easy to run in parallel, and high performance can be achieved by taking advantage of the fact that the filter responses for neighboring voxels use mainly the same input data. An easy way to implement a non-separable 3D convolution is to take advantage of the texture memory, as the texture memory cache can be used to speedup reads that are spatially local. Such an implementation will, however, be limited by the global memory bandwidth. A better approach is to take advantage of the local memory^8^ available in modern GPUs (CPUs do not normally have local memory physically; it can instead be simulated by the OpenCL driver). By first reading values from global memory into local memory, all the threads in a thread block can repeatedly read from the local memory very efficiently. The Nvidia GTX 680 has 48 KB of local memory per multiprocessor; it can for example store a 3D array of 32 × 32 × 12 float values. The quadrature filters used in BROCCOLI contain 7 × 7 × 7 coefficients, only 26 × 26 × 6 = 4,056 filter responses will therefore be valid for each multiprocessor. The reason for this is that the convolution is undefined along a boundary of (*N* − 1)/2 pixels for an N × N kernel. The yellow pixels in Figure 2 illustrate the invalid filter responses along the image borders for a filter size of 7 × 7. To maximize the number of valid filter responses per multiprocessor, a better approach to non-separable 3D convolution is to instead perform non-separable 2D convolution on the GPU, and then accumulate the filter responses by calling the convolution kernel for each slice of the filter [i.e., instead of running all 6 for-loops (three for the data and three for the filter) on the GPU, run 5 on the GPU and 1 on the CPU]. The local memory can for 2D be used to store two arrays of 96 × 64 float values, which instead gives a total of 10,440 valid filter responses per multiprocessor (two blocks of 90 × 58 pixels). The reason for using two arrays instead of one, is that each multiprocessor on the Nvidia GTX 680 can concurrently run 2048 threads, but only 1024 threads per thread block. The 1024 threads per block are arranged as 32 along the x-direction and 32 along the y-direction, to for example fit the number of local memory banks (32). Each thread starts by reading 6 values from global memory into local memory (96 × 64 / 1024 = 6) and then calculates 2 filter responses (giving two 32 × 32 blocks). Three additional filter responses are then calculated by most of the threads, yielding two blocks of 32 × 26 pixels and one block of 26 × 32 pixels. Finally, a number of threads are used to calculate the final 26 × 26 filter responses. The usage of local memory for non-separable 2D convolution is illustrated in Figure 2. As several quadrature filters need to be applied to the two volumes being registered (3 for linear registration and 6 for non-linear registration), 3 filters are applied simultaneosly once the data has been loaded into local memory. To achieve maximum performance, the for-loops have been unrolled manually using a Matlab script. To run a short for-loop on a GPU can result in a sub-optimal performance, as it can take a longer time to setup the for-loop than to run it (this is especially true for nested for-loops). The filters are stored in constant memory, as they are used by all threads and since each multiprocessor has a constant memory cache. The filter responses are stored in thread specific registers. Note that calculating 6 filter responses per thread results in a much better ratio of memory operations and calculations, compared to a straight forward approach using texture memory (where each thread calculates a single filter response). Interested readers are referred to our previous work (Eklund and Dufort, 2014) and our separate github repository^9^ for further details. The AMD GPU and the Intel CPU used in our case have only 32 KB of local memory, the AMD GPU can also only run 256 threads per thread block. The code for these devices instead uses one local memory array of 128 × 64 pixels and calculates 120 × 58 filter responses in blocks of 16 × 16 pixels.

The linear registration algorithm involves a summation over all voxels to setup an equation system (equation 6). BROCCOLI performs this summation using three kernels. The first kernel performs all the necessary multiplications and each thread calculates the sum for one voxel along the x-direction. The number of threads per thread block is equal to the width of the volume. The second kernel continues the summation along the y-direction (the number of threads per block is set to the depth of the volume) and the third kernel sums along z. The resulting equation system is finally copied to the host, to calculate the best parameter vector.

Except for the filtering and the summation operation, the other required functions are straight forward to implement. For the linear registration algorithm, one kernel is used in BROCCOLI to calculate phase differences (equation 9) and certainties and three kernels are used to calculate phase gradients ∇φ along x, y and z (Eklund et al., 2010). For the non-linear registration algorithm, one kernel is used to calculate the tensor components (equation 11), one kernel is used to setup the equation system in each voxel and one kernel solves the equation system (equation 12). Both the linear and the non-linear registration algorithm use one additional kernel to interpolate from the volume being moved to match the template. The texture memory is used for these two kernels, as it has hardware support for linear interpolation in 1, 2, and 3 dimensions. For all these kernels, each thread performs the operations for one voxel. To make sure that the same code runs on both Nvidia and AMD GPUs, 256 threads per block are used.

#### 2.6.2. Smoothing

The smoothing operation is also implemented as a convolution. As the Gaussian smoothing filters are Cartesian separable, three kernels are used to smooth along x, y, and z. Similarly to the non-separable convolution, local memory is used to obtain a more efficient implementation. The details of how the separable smoothing is performed will therefore not be given here.

#### 2.6.3. Statistical analysis

The statistical analysis of fMRI data is perfect for parallel processing; each thread performs the required calculations for one voxel. Just as for the registration kernels, all the statistical kernels use 256 threads per block to fit both Nvidia and AMD GPUs. For first level analysis assuming independent errors and for second level analysis, the pseudo inverse of the design matrix [i.e., (***X***^*T*^ ***X***)^−1^ ***X***^*T*^] is calculated on the host and stored in constant memory (as it is the same for all voxels). Calculation of the beta weights for one voxel can then be simply performed as a number of scalar products between the rows of the pseudo inverse and the data points of the current voxel (see equation 15). The resulting beta weights are stored as registers in each thread. However, each GPU thread can only handle a limited number of variables, BROCCOLI currently therefore supports a maximum of 25 regressors. To simply loop over the number of regressors may result in suboptimal performance, for two reasons. The first reason is that if the index to the beta array is not known at compile time, e.g., beta[i], the compiler may put beta in global memory instead of registers. The second reason is that short for-loops are inefficient on GPUs (as mentioned in the filtering implementation). For optimal performance, BROCCOLI instead uses a switch-case approach to first determine the number of regressors being used. The code for each case is also unrolled, such that all accesses to the beta array are known at compile time. To calculate the *t*-test or *F*-test value efficiently in each voxel, some additional values, e.g., ***c***^*T*^(***X***^*T*^ ***X***)^−1^ ***c*** from equation 16, are also pre-calculated and stored in constant memory. A limitation of the described approach is that the constant memory is normally only 32–128 KB; it can thus not store arbitrary large design matrices. A potential solution to this problem is to instead use texture memory, and take advantage of the texture memory cache instead of the constant memory cache.

The Cochrane-Orcutt procedure is harder to implement, as each voxel then uses a specific design matrix (after whitening according to equation 20). To calculate a pseudo inverse in each thread is problematic, as a design matrix for first level analysis easily can contain 200 timepoints and 15 regressors. Such an operation would thus require at least 3000 floats per thread, far outstripping the capabilities of some contemporary devices. For example, the Nvidia GTX 680 can handle only 63 floats per thread in its registers. Additional floats will spill into slow global memory (called local memory in CUDA), which may degrade the performance significantly. GPUs that have a L1 and/or L2 cache may be able to still use a larger number of registers efficiently. A possible solution could be to instead use the updating formula derived for MCMC (equation 24), but such an approach can also require a large number of registers [e.g., 40 registers for the ***m***_*ij*_ variables for 10 regressors and an AR(1) model]. The current solution is to instead calculate all the pseudo inverses on the host and then copy them to slow global memory. For these reasons, the Cochrane-Orcutt procedure is not yet optimized in terms of speed. Permutation testing for first level analysis therefore currently uses the simpler approach assuming independent errors. The permutation based *p*-values will still be valid, as the same analysis is applied in each permutation (whitening is applied prior to the permutations, and the autocorrelation is then put back in each permutation).

The whitening operation that is applied prior to the single subject permutations, and in the Cochrane-Orcutt procedure, requires that an AR model is estimated for each voxel. To accomplish this, each thread loops over time and sets up the Yule-Walker system of equations for one voxel. The AR(4) parameters are then calculated by directly solving these equations using a matrix inverse. One limitation of this approach is that more advanced AR models [e.g., an AR(8) model] requires a larger number of registers, both to store the parameters and to calculate the matrix inverse. For the inverse whitening applied in each permutation, to generate new data, all the threads also loop over time to generate new time series.

Permutation tests involving cluster based inference require that a clustering operation is performed in each permutation, to calculate the extent or mass of the largest cluster. BROCCOLI uses the parallel label equivalence algorithm proposed by Hawick et al. (2010) for this purpose. The algorithm is implemented as five kernels. The first kernel assigns an unique starting label to each voxel that survives the initial voxel-wise threshold (e.g., *p* = 0.01, uncorrected for multiple comparisons). In the second kernel each voxel checks its 26 neighbors to see if there is a label with a lower value. If a lower label is found, the label of the center voxel is updated and an update flag is set to 1. The third kernel resolves label equivalences, in order to minimize the number of times the second kernel has to be launched [see Hawick et al. (2010) for details]. The second and third kernels are launched repeatedly, until the update flag is no longer set to 1. To calculate the size of each cluster, a fourth kernel is applied where each thread atomically increments a cluster specific counter (determined by the cluster label). Finally, a fifth kernel is used to obtain the size of the largest cluster; the implementation relies on the atomic max operation.

The Bayesian MCMC algorithm can with careful memory management lead to a substantial time reduction compared to a sequential approach. To see the importance of memory management, consider simulating from the full conditional posterior of β and σ^2^. Conditional on ρ, this is a standard linear regression update on the transformed model

(23) $$\tilde{y}=\tilde{X}\tilde{\beta }+\tilde{\epsilon },$$

where $\tilde{X}$ and $\tilde{y}$ are obtained by pre-whitening ***X*** and ***y*** with the most recently simulated coefficients in ρ (as described in equations 19 and 20). Since ρ changes in every ρ-update, both $\tilde{X}$ and $\tilde{y}$ need to re-computed in each iteration of the Gibbs sampler. Both ***X*** and ***y*** are, however, too large to be stored in the fastest GPU memory (thread specific registers), and the cost of repeatedly accessing data from slower memory can be very large. To solve this problem, BROCCOLI instead updates ${\tilde{X}}^{T}\tilde{X}$ after a change in ρ, according to

(24) $${\tilde{X}}^{T}\tilde{X}=\sum_{i=0}^{p}\sum_{j=0}^{p}\rho_{i}\rho_{j}S_{ij},$$

where we for convenience define ρ_0_ = −1, *p* is the order of the AR model and $S_{ij}=\sum_{t=1}^{N}x_{t-i}x_{t-j}^{T}$ are data matrices independent of ρ (***x***_*t*_ is a vector that contains all the regressors for time point *t*, while ***X*** is the full design matrix). Note that ***S***_*ij*_ = ***S***_*ji*_ and that all ***S***_*ij*_ are symmetric. For a first order AR model, the update is given by

(25) $${\tilde{X}}^{T}\tilde{X}=S_{00}-2\rho S_{01}+\rho^{2}S_{11}.$$

Note how the data matrices ***S***_*ij*_ are separated from ρ in the above expressions. The ***S***_*ij*_ matrices are not voxel-specific and can, therefore, be pre-computed and stored in constant memory. Analogous formulas are easily derived for ${\tilde{X}}^{T}\tilde{y}$ (with data moments $m_{ij}=\sum_{t=1}^{N}x_{t-i}y_{t-j}$) and ${\tilde{y}}^{T}\tilde{y}$ (with data moments $g_{ij}=\sum_{t=1}^{N}y_{t-i}y_{t-j}$), both of which are needed for the Gibbs sampling. The ***m***_*ij*_ and the *g*_*ij*_ values are voxel-specific, but low-dimensional and can therefore be stored in thread specific registers. Despite these optimizations, the implementation can currently only handle a small number of regressors and an AR(1) model of the residuals. The extension to more elaborate models is in principle straight forward however, and rapid advancements in GPU memory are likely to remove these limitations in the near future

The Bayesian fMRI analysis also requires random number generation to estimate the joint posterior distribution. The CURAND library can be used for this purpose for the CUDA programming language, but there exists no similar library for OpenCL. Instead, random numbers are first generated in BROCCOLI from a uniform distribution, using a voxel/thread specific seed and a modulo operation (Langdon, 2009). This is the only part of the OpenCL code that currently uses double precision. The seeds are generated on the host side, as this operation only needs to be performed once. The uniformly distributed numbers are then used to generate numbers from a normal distribution, by applying the Box-Muller transform (Box and Muller, 1958). Random numbers from an inverse Gamma distribution can finally be generated as

(26) $$g=\frac{2B}{\sum_{i=1}^{2A}n_{i}^{2}},$$

where *n* is a random number from a normal distribution with zero mean and unit variance, *A* is the shape parameter of the Gamma distribution and *B* is the scale parameter.

## 3. Results

A number of freely available fMRI datasets (Biswal et al., 2010; Poldrack et al., 2013) were used to test our software, and to compare it to existing software packages. The hardware used for testing is specified in Table 1. Specifically, BROCCOLI was used with an Intel CPU, an Nvidia GPU and an AMD GPU, to demonstrate that the same code can run on different types of hardware. The following software packages were compared to BROCCOLI: SPM8, FSL 5.0.4 (Smith et al., 2004) (with the package Condor installed for parallel processing) and AFNI (Cox, 1996) (with OpenMP support for parallel processing). For FSL, the shell variable FSLPARALLEL was set to “condor” to measure multi-core results. For AFNI, the shell variable OMP_NUM_THREADS was set to “1” to generate processing times for single-core processing, and to “8” for multi-core processing. BROCCOLI running on a CPU automatically uses all available processor cores for all processing steps. All testing scripts can be downloaded from github^10^. To make the comparison reflective of each package's standard use, our testing scripts were posted on the mailing lists for SPM, FSL, and AFNI and modified according to responses.

It should be stressed that the different software packages use different algorithms, programming languages and libraries. It is therefore hard to make a quantitatively meaningful performance comparison. For this reason, we also added the processing time for BROCCOLI running on a single CPU core, such that there is a baseline comparison for each algorithm. This was achieved by setting the shell variable CPU_MAX_COMPUTE_UNITS to 1 (a more general and complicated way is to use OpenCL device fission).

### 3.1. Spatial normalization

The quality of the normalization to MNI space was tested by aligning 198 T1-weighted volumes to the MNI brain templates (1 and 2 mm^3^ resolution) provided in the FSL software (MNI152_T1_1 mm_brain.nii.gz, MNI152_T1_2 mm_brain.nii.gz). The T1 volumes were downloaded from the 1000 functional connectomes project (Biswal et al., 2010), and the Cambridge dataset was selected for its large number of subjects. Each T1 volume is of the size 192 × 192 × 144 voxels with a resolution of 1.2 × 1.2 × 1.2 mm. To fully focus on the registration algorithm, the provided skullstripped T1 volumes were used rather than the original T1 volumes.

For SPM the functions “Normalize” and “Segment” were used for normalization. For “Normalize,” the parameter 'Source image smoothing' was changed from 8 mm to 4 mm, to try to match the smoothness of the FSL T1 template (the T1 template in SPM is more blurred than the T1 template in FSL). For 'Segment', an initial parametric alignment of each T1 volume was first performed using the function 'Coregister' (otherwise several normalized T1 volumes were far off from the MNI template). Except for these modifications, the default settings were used. For FSL, the T1 volumes were aligned by running FLIRT (which performs linear registration) using the skullstripped volume and template, followed by FNIRT (which performs non-linear registration) using the volume and template with skull (this is the recommended usage). The estimated deformation field was finally applied to the skullstripped volume. For registration to the 2 mm^3^ MNI template, the configuration file “T1_2_MNI152_2 mm.cnf” was used, while the default settings were used for registration to the 1 mm^3^ template (there is no “T1_2_MNI152_1 mm.cnf”). For AFNI, alignment was performed correspondingly by running 3dUnifize (which normalizes the image intensity) both for the T1 volume and the MNI template, 3dAllineate and 3dQwarp. The estimated displacement field was finally applied to the original T1 volume without intensity normalization, using the function 3dNwarpApply. The default interpolation method for 3dNwarpApply is sinc interpolation, but as SPM, FSL and BROCCOLI all use linear interpolation by default, 3dNwarpApply was tested with linear as well as sinc interpolation. The non-linear registration in 3dQwarp is done with a combination of cubic and quintic basis functions, and it is not possible to change this to linear interpolation. Since 3dQwarp in AFNI is a very new method, we used settings proposed in the help text. For all software packages, the same settings were used for each T1 volume.

**Figure 3:**
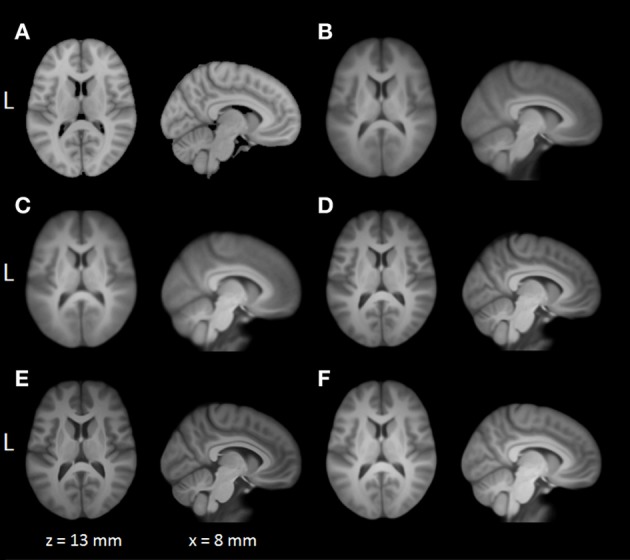
**A visual comparison of spatial normalization with the different software packages, by averaging 198 normalized T1 volumes. (A)** MNI template, **(B)** SPM Normalize average normalized T1 volume, **(C)** SPM Segment average normalized T1 volume, **(D)** FSL average normalized T1 volume, **(E)** AFNI average normalized T1 volume, **(F)** BROCCOLI average normalized T1 volume. Note that AFNI uses a combination of cubic, quintic, and sinc interpolation as default, while SPM, FSL, and BROCCOLI all use linear interpolation as default.

**Figure 4:**
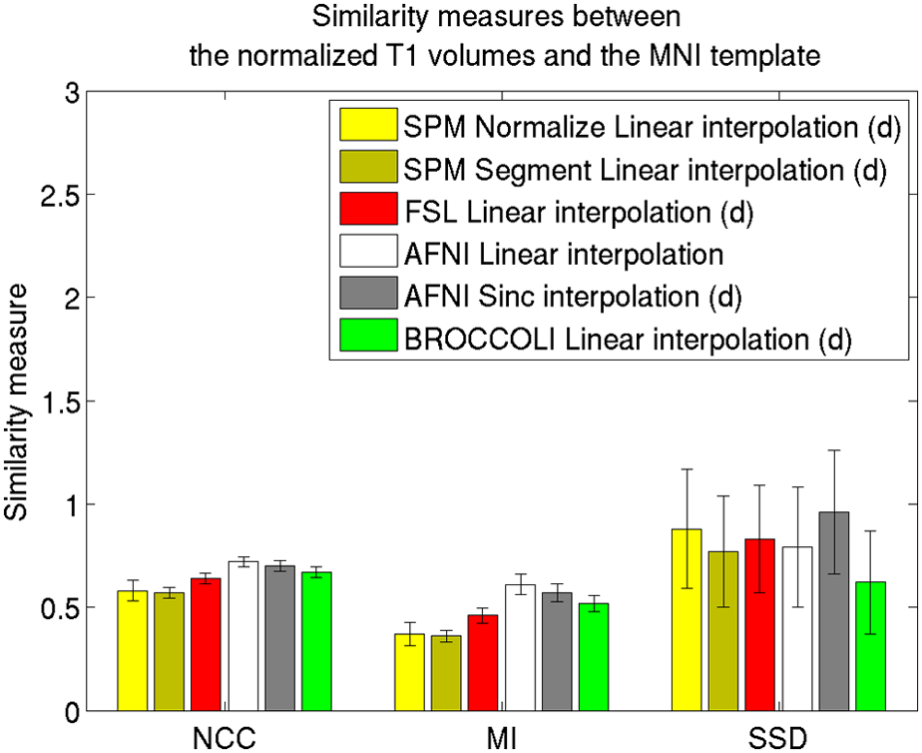
**Similarity measures between each normalized T1 volume and the MNI template for the different software packages, averaged over 198 subjects.** The error bars represent the standard deviation. NCC stands for normalized cross correlation (higher is better), MI stands for mutual information (higher is better) and SSD stands for sum of squared differences (lower is better). For visualization purposes, the SSD similarity measure was divided by 300,000. Only the voxels in the MNI brain mask were used to calculate these similarity measures, as 75% of the voxels are outside the brain. Different interpolation modes were tested, as the software packages have different default settings for interpolation (d denotes the default interpolation).

**Figure 5:**
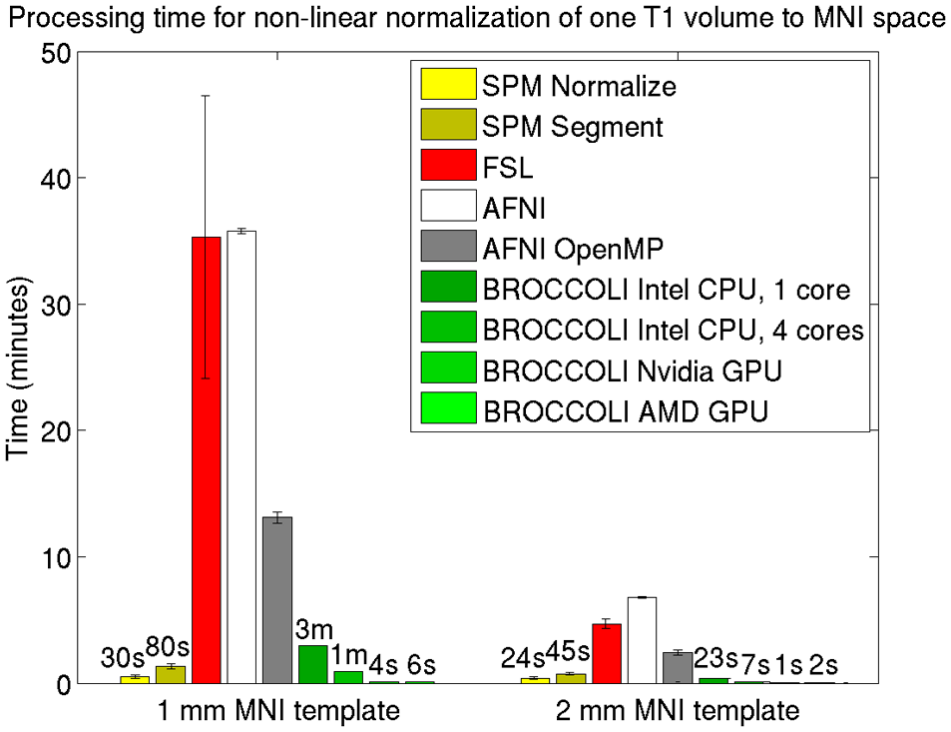
**Processing times for non-linear spatial normalization of one T1 volume of size 192 × 192 × 144 voxels to a MNI template (1 and 2 mm^3^ resolution) for the different software packages, averaged over 198 T1 volumes.** The error bars represent the standard deviation. Note that AFNI uses a combination of cubic, quintic, and sinc interpolation as default, while SPM, FSL, and BROCCOLI all use linear interpolation as default. A linear registration was first applied to achieve a good starting point for the non-linear registration. BROCCOLI running on a GPU can perform non-linear normalization to a 1 mm^3^ template in 4–6 s, and still provide a satisfactory result. BROCCOLI running on a CPU is also significantly faster than FSL and AFNI OpenMP, even if a single CPU core is used.

Average normalized T1 volumes were calculated for SPM, FSL, AFNI, and BROCCOLI, to visually compare the algorithms, and are given in Figure 3. It should be noted that for FSL, the resulting displacement field from the 2 mm^3^ normalization was upscaled and used to generate the normalized T1 volumes used here (as recommended by the FSL mailing list). For a more numerical comparison of the image registration quality, the normalized cross-correlation, mutual information and sum of squared differences were calculated between each normalized T1 volume and the MNI template, the mean results are given in Figure 4. Only the voxels inside the MNI brain mask were used to calculate these similarity measures, as 75% of the voxels are outside the brain. The processing time for the different software packages are given in Figure 5.

### 3.2. Motion correction

**Figure 6:**
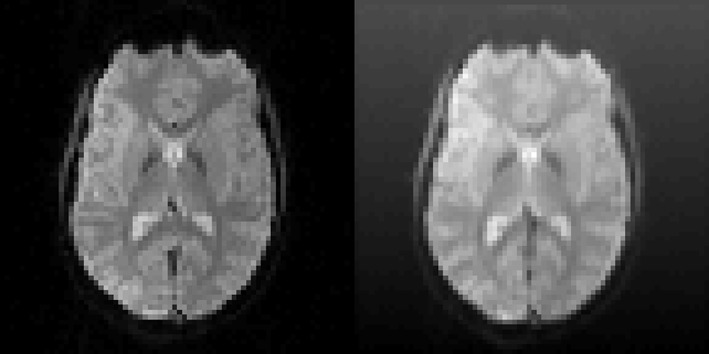
**Left:** One slice of one fMRI dataset used for testing the motion correction algorithms. **Right:** The same slice after the application of a random translation and rotation, and addition of a shading (gradient) increasing upwards. The shading will affect all algorithms that use the image intensity directly. The phase based algorithm used in BROCCOLI will, however, not be affected by this shading. The main reason for this is that quadrature filters are bandpass filters, which remove low frequency variations (e.g., shadings) as well as high frequency variations (e.g., noise).

The motion correction algorithms in SPM (realign), FSL (MCFLIRT), AFNI (3dvolreg), and BROCCOLI were tested by using test datasets with known motion parameters. The test datasets were generated by repeatedly using only the first fMRI volume in each dataset and applying known random rigid transformations to this first volume. The translations and rotations were independently generated from a normal distribution with a mean of 0 and a standard deviation of 0.5 (voxels for translations and degrees for rotations). Gaussian white noise was then added to each volume. To further demonstrate the robustness of BROCCOLI's phase based algorithm, a shading was added to each transformed fMRI volume. An example of the added shading is given in Figure 6. The test datasets were created using the 198 resting state datasets in the Cambridge dataset (Biswal et al., 2010). Each rest dataset is of the size 72 × 72 × 47 × 119 with a voxel resolution of 3 × 3 × 3 mm.

For SPM and AFNI, the algorithms were tested with linear interpolation in addition to the default setting (b-spline for SPM and Fourier for AFNI), as FSL and BROCCOLI use linear interpolation as default. For SPM and FSL, the reference volume was set to the first volume, which is the default for AFNI and BROCCOLI. Except for these changes, the default settings were used for all software packages. The quality of the motion correction was evaluated by comparing the estimated transformations to the true ones. For each dataset, the total error was calculated as the square root of the sum of the squared differences over all motion parameters *p* and time points *t*, i.e.,

(27) $$\epsilon =\sqrt{\sum_{t=1}^{119}\sum_{p=1}^{6}{\left(motion_{estimated}(t,p)-motion_{true}(t,p)\right)}^{2}}.$$

**Figure 7:**
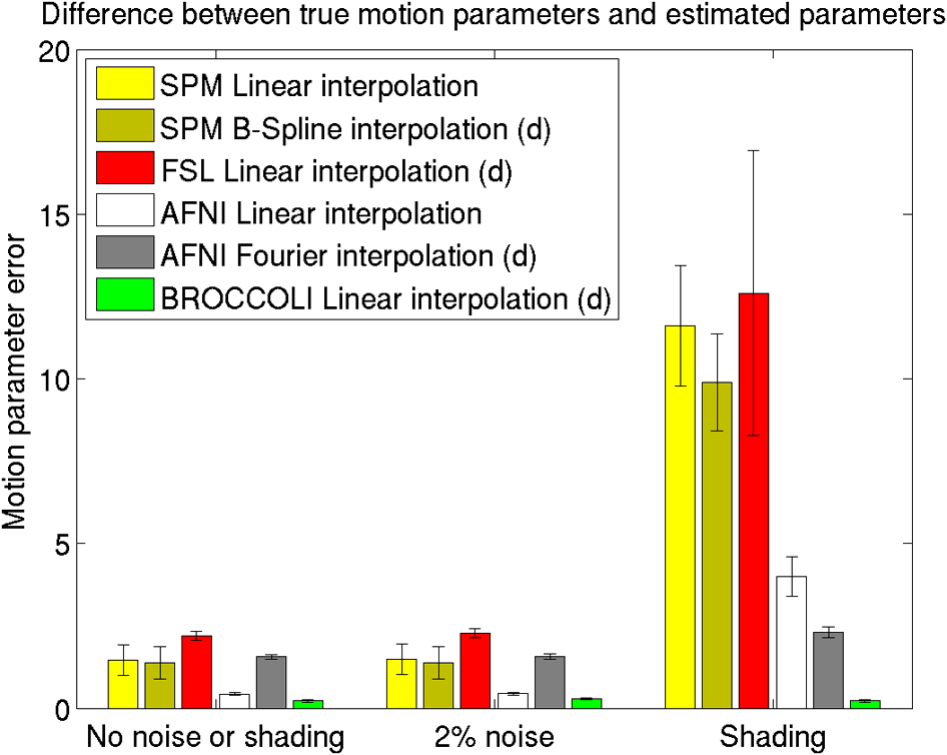
**Motion parameter errors for the different software packages, averaged over 198 datasets with artificial motion.** The error bars represent the standard deviation. The testing datasets were generated by applying random translations and rotations to the first fMRI volume in each dataset, and then adding Gaussian noise or a shading. The amount of noise was defined by setting the standard deviation to a percentage of the maximum intensity value. Different interpolation modes were tested, as the software packages have different default settings for interpolation (d denotes the default interpolation). The presented results were generated with an Nvidia GPU, and equal results were also obtained by the Intel CPU and the AMD GPU.

**Figure 8:**
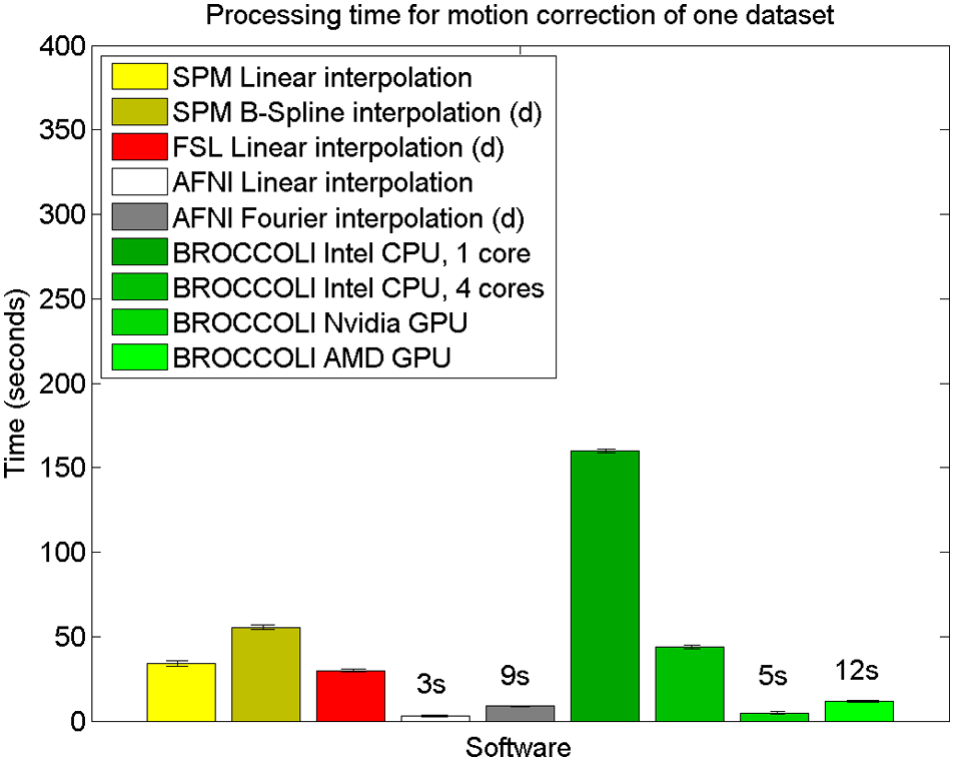
**Processing times for motion correction of one fMRI dataset of size 72 × 72 × 47 × 119 for the different software packages, averaged over 198 datasets.** The error bars represent the standard deviation. All algorithms registered all volumes to the first one. The processing times for AFNI and AFNI OpenMP are the same, as the AFNI software does not have any OpenMP support for motion correction. Different interpolation modes were tested, as the software packages have different default settings for interpolation (d denotes the default interpolation).

The mean error measures for the different software packages, averaged over the 198 subjects, are given in Figure 7 and the processing times for motion correction are given in Figure 8.

### 3.3. First level analysis

The first level analysis was tested by analyzing freely available task fMRI datasets, downloaded from the OpenfMRI (Poldrack et al., 2013) homepage. Specifically, the OpenfMRI “rhyme judgment” dataset was used where the subjects were presented with pairs of either words or pseudowords, and made rhyming judgments for each pair. See the work by Xue and Poldrack (2007) for further information about this dataset.

#### 3.3.1. Frequentist inference

**Figure 9:**
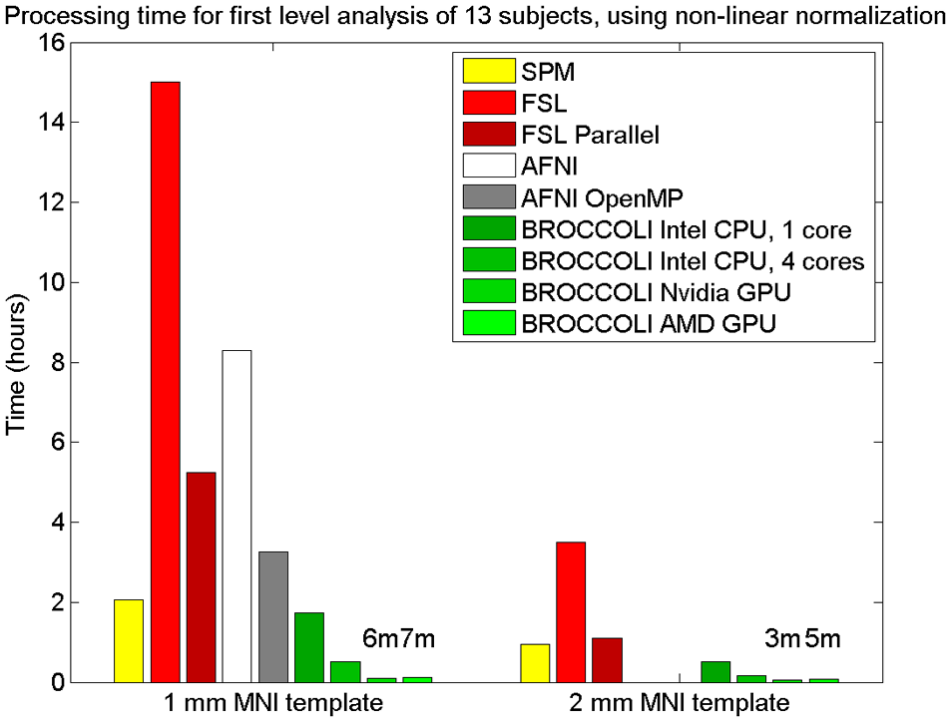
**Processing times for first level analysis of 13 fMRI datasets (of size 64 × 64 × 33 × 160).** The analysis includes non-linear normalization to a brain template, slice timing correction, motion correction, smoothing, and statistical analysis. A Matlab script, available on the SPM homepage, was used for SPM. For FSL, the analysis was setup and started through the graphical user interface. For AFNI, the analysis was performed with *afni_proc.py*, through the graphical user interface *uber_subject.py*. It should be noted that SPM, FSL and BROCCOLI use linear and non-linear registration, while AFNI uses linear registration only (currently, it is not possible to select non-linear registration in uber_subject.py). To compensate for this, the non-linear registration for AFNI was done separately. Note that it is not possible to select a 2 mm^3^ brain template in uber_subject.py, these processing times are therefore not defined. Also note that the processing times for BROCCOLI do not include any first level permutation test.

**Figure 10:**
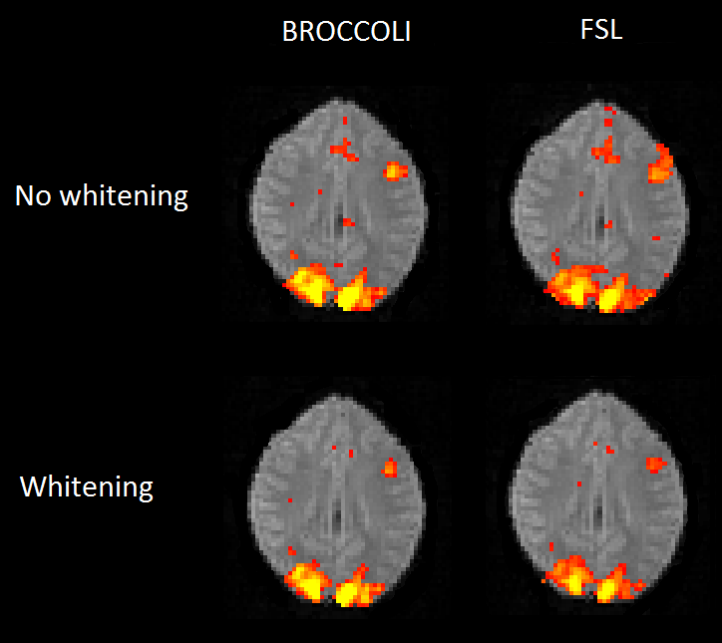
**Brain activity maps (representing *t*-values) from first level analysis of one OpenfMRI dataset, for BROCCOLI and FSL.** Subjects were presented with pairs of either words or pseudowords in a block based design, and made rhyming judgments for each pair. The first level analysis here includes motion correction, segmentation of the fMRI data, smoothing, and statistical analysis. Both BROCCOLI and FSL used motion regressors in the statistical analysis. As BROCCOLI and FSL use different models of the GLM residuals, we here present activity maps with and without whitening. The activity maps have been arbitrarly thresholded at a *t*-value of 5.

**Figure 11:**
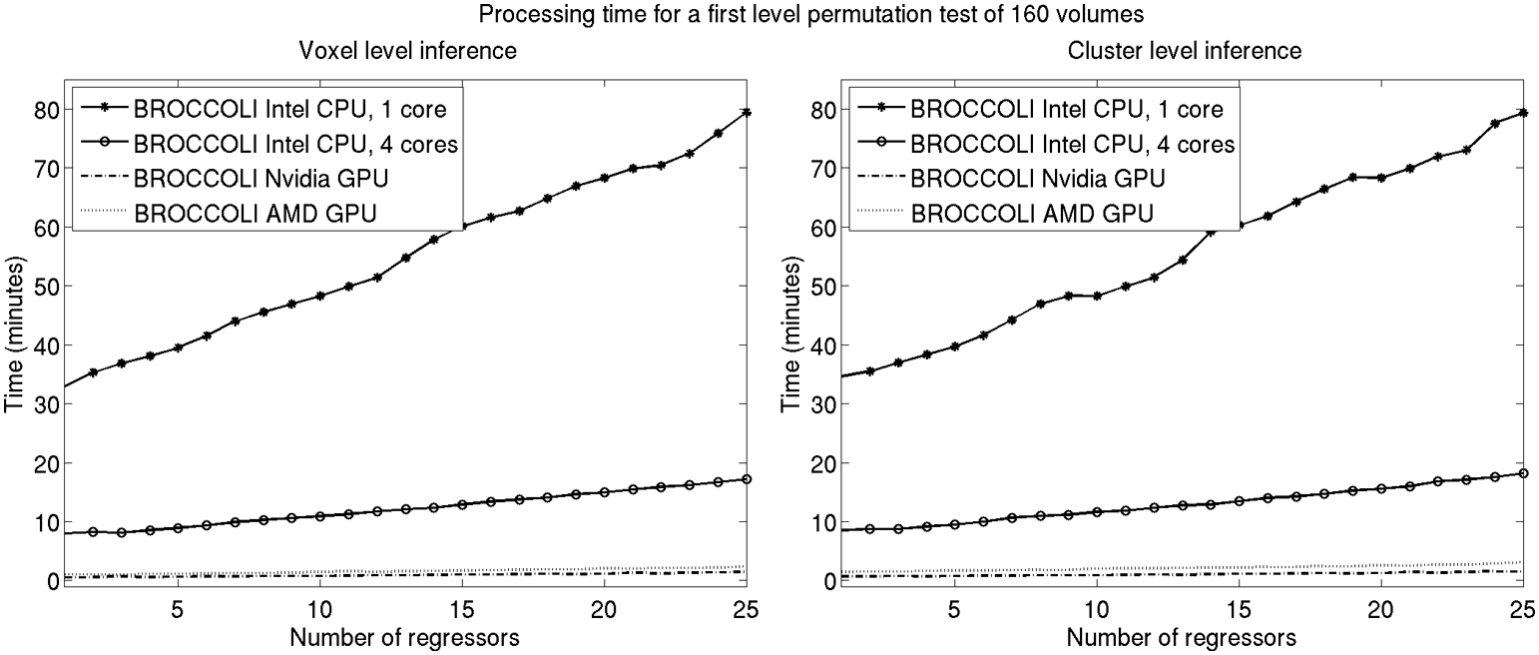
**Processing times for BROCCOLI for first level analysis using a permutation based *t*-test with 10,000 permutations (SPM, FSL, and AFNI do not provide any functions for first level permutation based analysis). Left:** Voxel-level inference, the maximum *t*-test value is saved in each permutation. **Right:** Cluster-level inference, the extent of the largest cluster is saved in each permutation. A *t*-value of 3 was used as a cluster defining threshold. The data used is of the size 64 × 64 × 33 × 160. A brain mask was used to only perform the statistical calculations for the brain voxels. Note that these processing times do not include smoothing in each permutation. Smoothing the fMRI data 10,000 times takes about 8970 s using one core on the Intel CPU, 2710 s using all the four cores on the Intel CPU, 335 s with the Nvidia GPU and 550 s with the AMD GPU. Also note that ordinary least squares is used to estimate the GLM beta weights in each permutation, and not the more demanding Cochrane-Orcutt procedure.

To the best of our knowledge, the SPM software package does not have any default processing pipeline. Instead, we used a batch script for first level analysis available on the SPM homepage^11^. For FSL, the analysis was setup and started through the graphical user interface. For AFNI, the Python script afni_proc.py was used, through the graphical interface uber_subject.py. The settings used for each software are given in Table 2. Processing times for first level analysis for the different software packages are given in Figure 9. A visual comparison of one brain activity map, for BROCCOLI and FSL, is given in Figure 10. Processing times for BROCCOLI for a first level permutation-based analysis, using 10,000 permutations, are given in Figure 11.

**Table 2:** **Settings for first level analysis for the different software packages (for AFNI it is currently not possible to select non-linear registration in the graphical user interface)**.

	**Normalization**	**Motion**	**Motion regressors**	**Smoothing (mm)**	**Cluster simulation**	**Modeling of GLM residuals**
SPM	Linear + non-linear to MNI template	Yes	Yes, 6	6	Not available	Global AR(1)
FSL	Linear + non-linear to MNI template	Yes	Yes, 6	6	Not available	FILM prewhitening (Woolrich et al., 2001)
AFNI	Linear to MNI template	Yes	Yes, 6	6	No	Voxel-wise ARMA(1, 1)
BROCCOLI	Linear + non-linear to MNI template	Yes	Yes, 6	6	Not available	Voxel-wise AR(4)

#### 3.3.2. Bayesian inference

**Figure 12:**
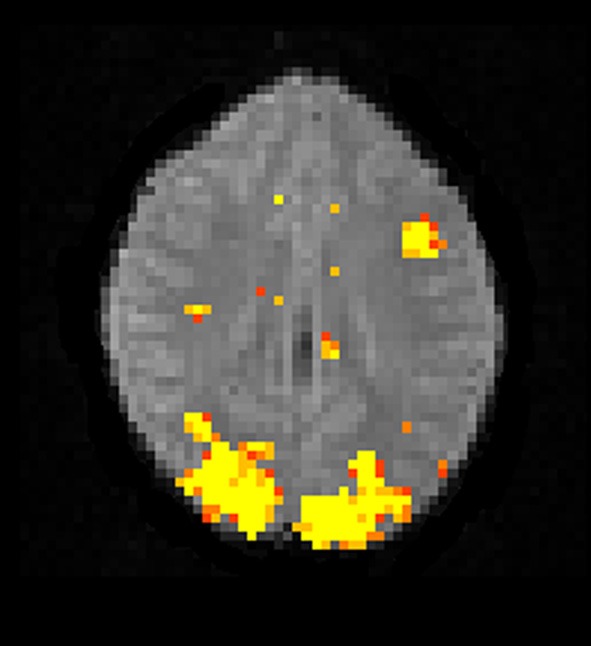
**A posterior probability map (PPM) from a Bayesian first level analysis of one OpenfMRI dataset.** Subjects were presented with pairs of either words or pseudowords in a block based design, and made rhyming judgments for each pair. The first level analysis here includes motion correction, segmentation of the fMRI data, smoothing, and statistical analysis. The PPM represents the probability of the first GLM beta weight being larger than zero, and has been arbitrarly thresholded at a probability of 0.99. Note that the PPM has been calculated by using a Gibbs sampler, and not by using techniques based on variational Bayes. Also note that the frequentist approach uses a voxel-wise AR(4) model of the GLM residuals, while the Bayesian currently uses a voxel-wise AR(1) model (due to hardware limitations).

The Bayesian fMRI analysis was tested by generating a total of 11,000 draws from the posterior distribution for each brain voxel (44,220 voxels), and the first 1000 draws were discarded as “burn in” samples. The PPM was calculated as the percentage of draws where the GLM weight of interest was larger than zero. The resulting PPM is given in Figure 12, and can be compared to the t-map in Figure 10. The processing time was 4706 s using the Intel CPU and one core, 835 s using the Intel CPU and all the four cores, 190 s for the Nvidia GPU and 91 s for the AMD GPU. This can be compared to about 20 h for a naive Matlab implementation.

### 3.4. Second level analysis

**Figure 13:**
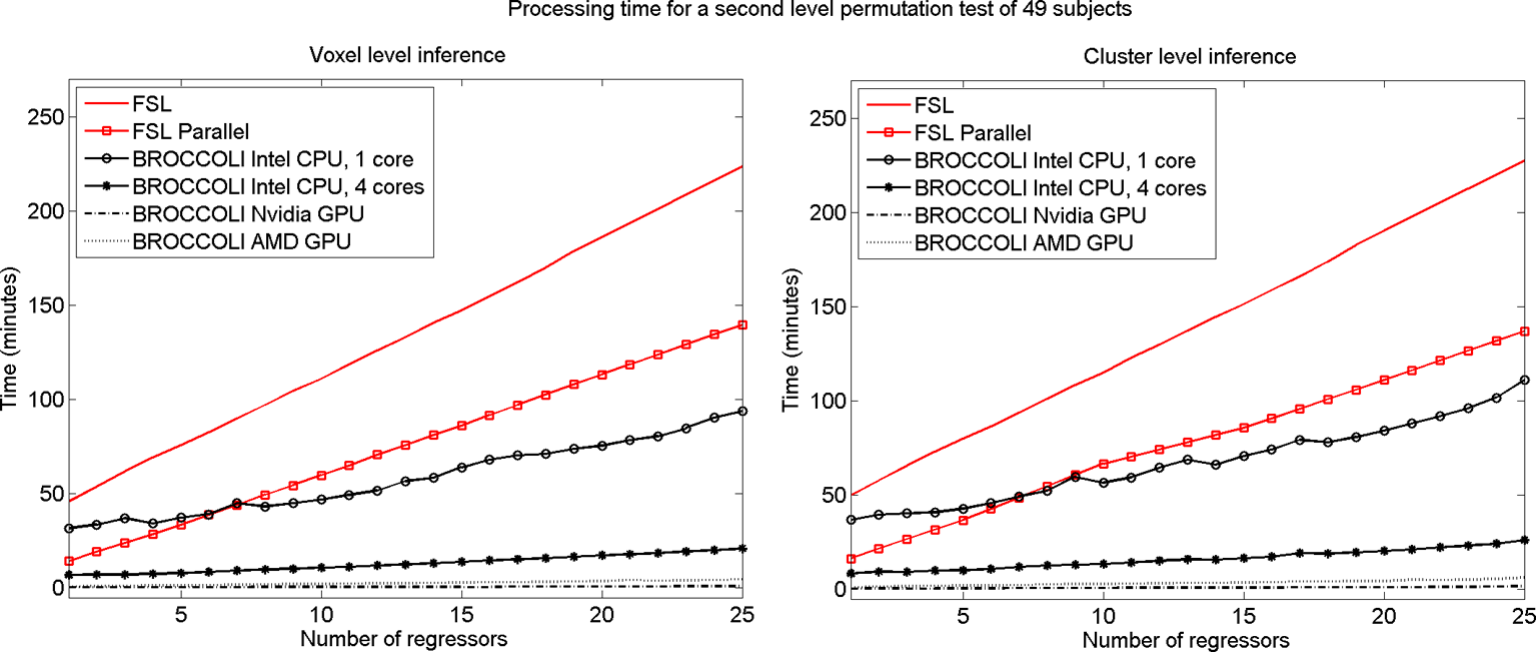
**Processing times for second level analysis using a permutation based *t*-test with 10,000 permutations, for BROCCOLI and FSL (the SPM and AFNI software packages do not provide any functions for permutation based analysis). Left:** Voxel-level inference, the maximum *t*-test value is saved in each permutation. **Right:** Cluster-level inference, the extent of the largest cluster is saved in each permutation. A *t*-value of 3 was used as a cluster defining threshold. The data used are beta volumes from 49 subjects, where each beta volume is of the size 91 × 109 × 91 voxels. A brain mask was used to only perform the statistical calculations for the brain voxels. The processing time for FSL increases quickly with the number of regressors, while the processing time for BROCCOLI increases much more slowly. This is explained by the fact that calculations on a GPU are efficient, once all the data have been loaded from the slow global memory to the fast thread specific registers. To estimate several beta weights per voxel, instead of a single weight, therefore results in a better utilization of the GPU performance. The processing time for BROCCOLI using an AMD GPU is 2–5 times as high compared to BROCCOLI using a Nvidia GPU. One possible explanation for this is that the code was converted from CUDA to OpenCL. Note that these processing times are for data normalized to a 2 mm^3^ MNI template. The permutation tests would take approximately eight times longer for data normalized to a 1 mm^3^ MNI template.

To test the second level analysis, the permutation functionality in BROCCOLI was compared to the function randomize in FSL (SPM and AFNI do not have any support for permutation based analysis, although AFNI for example has support for Kruskal-Wallis tests and Wilcoxon tests). The function randomize_parallel in FSL automatically divides the number of permutations to a number of computers or CPU cores (if for example Condor or GridEngine is installed), and was therefore also used for testing. First level results generated by FSL (downloaded from the OpenfMRI homepage) were used as inputs to the second level analysis, to fully focus on the permutation procedure. Here we used the OpenfMRI dataset “word and object processing”, as it has the largest number of subjects (49). See the work by Duncan et al. (2009) for further information about this dataset. Processing times for FSL and BROCCOLI for a second level permutation-based analysis of the 49 subjects, using 10,000 permutations, are given in Figure 13. Null distributions generated by FSL and BROCCOLI, for a design matrix containing a single regressor, were compared numerically and were found to be equivalent. A direct comparison for more than one regressor is more problematic, as the randomize function in FSL first transforms the design matrix to effective regressors and effective confound regressors, by using information from the contrast vector.

## 4. Discussion

We have presented a new software package for fMRI analysis. BROCCOLI is written in OpenCL, making it possible to run the analysis in parallel, taking full advantage of a large variety of hardware configurations. To exemplify this, BROCCOLI has been tested with an Intel CPU, an Nvidia GPU and an AMD GPU. The main objective of BROCCOLI is to demonstrate the advantages of parallel processing and to enable the neuroimaging field to avail itself of more computationally demanding normalization algorithms, and statistical methods that are based on a smaller number of assumptions (e.g., by using non-parametric statistics). Currently, BROCCOLI reduces the fMRI processing time by at least an order of magnitude compared to existing software packages (even if only a CPU and not a GPU is used). For non-linear spatial normalization, BROCCOLI running on an Nvidia GPU is approximately 525 times faster compared to FSL and AFNI, and 195 times faster than AFNI OpenMP. For second level permutation tests, BROCCOLI using an Nvidia GPU is 100–200 times faster than FSL and 33–130 times faster than the parallel version of FSL.

### 4.1. Spatial normalization

The accuracy measures illustrated in Figures 3 and 4 reveal a number of interesting differences. The normalization in AFNI yields the highest mean correlation and mutual information. It might seem non-intuitive that the sinc interpolation in AFNI gives a higher sum of squared differences compared to the linear interpolation, but this is possibly explained by the fact that the sinc interpolation preserves high resolution details, perhaps beyond the meaningful resolution of the MNI template. The average normalized T1 volumes generated by SPM are clearly the most blurred, although the algorithms are fast compared to FSL and AFNI. The results presented here are consistent with a previous comparison (Klein et al., 2009), where the FSL function FNIRT was shown to provide better normalizations than the SPM functions “Segment” and “Normalize.” AFNI was not included in this comparison, as the function 3dQwarp was released recently.

These comparisons should not be considered as a thorough head-to-head evaluation of the different software packages. Rather, the motivation was to show that BROCCOLI can provide a satisfactory normalization to MNI space in a short amount of time. An aspect not considered here, for example, is the smoothness of the resulting displacement fields. It is also possible that the different algorithms would perform better if the default settings were changed.

### 4.2. Motion correction

The evaluation of the motion correction algorithms shows that BROCCOLI yields the smallest difference between the true motion parameters and the estimated ones, closely followed by AFNI. BROCCOLI using a GPU and AFNI perform the motion correction in a similar amount of time, while SPM and FSL are significantly slower. For BROCCOLI running on a CPU, the processing time is rather long, which is mainly explained by the fact that three (non-separable) quadrature filters need to be applied for each time point and for each iteration (3–5 iterations of the linear registration algorithm is normally sufficient for motion correction). BROCCOLI also estimates 12 affine registration parameters for each time point, and then restricts them to a rigid transformation (6 parameters). The results presented here are consistent with a previous comparison of motion correction algorithms (Oakes et al., 2005), where the AFNI software was shown to provide the most accurate motion estimates.

It should be noted that the test used here is not based on realistic head motion, as completely random transformations were applied for each time point. This can, for example, negatively effect the MCFLIRT function used in FSL. The reason for this is that MCFLIRT uses the motion estimate from the previous time point as a starting estimate for the next time point (Jenkinson et al., 2002). Similarly, 3dvolreg in AFNI is only intended for small motions, and the transformations applied here may have been too severe. The shading test is also not very realistic, but clearly shows the robustness of phase based registration algorithms compared to intensity based algorithms. For these reasons, the presented results should be interpreted with caution.

### 4.3. First level analysis

#### 4.3.1. Frequentist inference

The first level analysis using FSL and BROCCOLI yield very similar results, both with and without pre-whitening to correct for auto correlation in the GLM residuals. The small differences in activation between FSL and BROCCOLI can be explained by a number of factors. The motion correction algorithms, for example, provide slightly different results according to Figure 7 and this will affect further processing. There are also some differences in how FSL and BROCCOLI setup the design matrix and treat the auto correlation of the GLM residuals. BROCCOLI uses four detrending regressors (mean, linear trend, quadratic trend, cubic trend) while FSL instead applies a temporal filtering to the data and the regressors. BROCCOLI smooths all the AR estimates in the same way, while FSL separately smooths AR estimates in white and gray brain matter (Woolrich et al., 2001).

BROCCOLI is significantly faster than SPM, FSL, and AFNI, even when the analysis is run on a CPU. SPM is also faster than FSL and AFNI, which is mainly explained by a faster spatial normalization. The parallel version of FSL, where one first level analysis in our case runs on each CPU thread, is significantly faster than the non-parallel version. However, as the first level analysis in FSL requires more than 2 GB of memory, we were only able to run 6 (instead of 8) threads in parallel (since the computer used for testing has 16 GB of memory).

#### 4.3.2. Bayesian inference

The Bayesian first level analysis yields results that are similar to the *t*-maps, although the results cannot be compared directly. It might seem confusing that the AMD GPU is faster than the Nvidia GPU, especially since the Nvidia GPU is faster for permutation tests. The reason for this is that the random number generation currently uses double precision, and the AMD GPU used in our case has better support for such calculations than the Nvidia GPU (see Table 1).

### 4.4. Second level analysis

The processing times in Figure 13 for the second level permutation test may at first appear confusing. The speedup of using randomize_parallel instead of randomize decreases with the number of regressors, from a speedup of 3.2 for a single regressor to 1.6 for 25 regressors (but the actual time saved increases). The 10,000 permutations are divided into smaller work items of 300 permutations each for randomize_parallel. However, 33 work items cannot be divided equally to a CPU running 8 threads (8 threads ∗ 4 work items per thread = 32 work items). The permutation test is therefore not completed until the last work item has been processed, for which only a single CPU thread is active. The unequal division is more problematic for more regressors, as each work item then takes a longer time to process.

The processing time for BROCCOLI is not affected as much by the number of GLM regressors as the FSL software is, resulting in a larger speedup for a larger number of regressors. A GPU thread that performs a small number of calculations is very limited by the memory bandwidth. More regressors lead to more calculations and, thereby, a better utilization of the computational capabilities of a GPU. BROCCOLI running on a CPU is also faster than the parallel version of FSL. FSL divides the work into several CPU cores by using a package like Condor or GridEngine. Such an approach cannot as easily take advantage of vectorized operations [e.g., Intel streaming SIMD extensions (SSE)], where the same operation is applied to a number of elements simultaneously. Note that this is a distinct, second layer of parallel processing. In addition to the code running on several CPU cores instead of just one, the processing on each individual core is vectorized, performing 4–16 arithmetic operations on different data at once.

It should also be noted that the presented processing times are for fMRI data registered to a 2 mm^3^ MNI template, each permutation test would take approximately 8 times longer for data registered to a 1 mm^3^ MNI template. Threshold free cluster enhancement (Smith and Nichols, 2009) is another inference method that would benefit from GPU acceleration, as it is much more computationally demanding compared to voxel-level and cluster-level inference.

### 4.5. Limitations

The following list itemizes the current limitations of using BROCCOLI:

- BROCCOLI currently has very limited support for image segmentation, but such algorithms are often easy to run in parallel (Eklund et al., 2013a).
- The quality of the fMRI-to-T1 registration has not been tested as extensively as the T1-to-MNI registration. There are, at least, two reasons why the fMRI-to-T1 registration is harder to test than the T1-to-MNI registration. First, the fMRI data is of much lower spatial resolution and an average of 198 registered fMRI volumes would therefore be extremely blurry. Second, the fMRI data is often distorted due to artifacts from the MRI sequence.
- The SPM, FSL, and AFNI software packages have been used for a long time and have been extensively tested, while BROCCOLI is completely new software.
- SPM, FSL, and AFNI all provide a graphical user interface, which BROCCOLI currently does not.
- SPM, FSL, and AFNI all provide a large number of functions which can be combined to basically solve any problem. BROCCOLI is on the other hand currently limited to image registration and first and second level fMRI analyses.
- SPM, FSL, and AFNI all provide some sort of community forum where users can get help.

### 4.6. Future work

In the future, BROCCOLI can be improved and extended in several ways. The most important addition may be a graphical user interface, so that as many researchers as possible can take advantage of parallel processing. For the first version of BROCCOLI we have focused on functionality and stability, and not so much on the computational performance. As most of the code was converted from CUDA to OpenCL, it is likely that BROCCOLI performs best for Nvidia GPUs. Optimizing the code for other hardware platforms (e.g., Intel and AMD) will therefore be one important project (Enmyren and Kessler, 2010). For permutation tests involving large datasets, multi-GPU support can be used to further reduce the computational burden, by running a number of permutations on each GPU (Eklund et al., 2011a). First level analysis can also run in parallel on several GPUs with multi-GPU support, such that each GPU independently processes one subject. Another natural extension would be to provide several other wrappers for BROCCOLI, such as R and bash.

Rather than using ordinary least squares to estimate beta weights in the GLM, it would be interesting to, for example, use a regularized regression approach such as LASSO (Tibshirani, 1996) instead. LASSO is often used together with cross validation, and would be rather time consuming to run for every voxel. This is especially true if LASSO is combined with a permutation procedure, to correct for multiple comparisons. Most fMRI researchers use the GLM for the statistical analysis, but multivariate approaches that adaptively combine timeseries of several voxels can, in some cases, yield higher statistical power. We would therefore also like to convert our existing CUDA code for canonical correlation analysis (CCA) (Friman et al., 2003; Eklund et al., 2011a) to OpenCL and include it in BROCCOLI. The null distribution of canonical correlations is much more complicated than conventional *t*-tests, a problem which can be solved with permutation-based procedures.

### Conflict of interest statement

The authors declare that the research was conducted in the absence of any commercial or financial relationships that could be construed as a potential conflict of interest.

## Footnotes

^1^http://fcon_1000.projects.nitrc.org/fcpClassic/FcpTable.html

^2^https://openfmri.org/

^3^http://www.humanconnectome.org/

^4^https://github.com/wanderine/BROCCOLI/

^5^http://eigen.tuxfamily.org/index.php?title=Main_Page

^6^https://bitbucket.org/eigen/eigen/

^7^For readers not familiar with GPU programming, the CPU is often called the host while the GPU is called the device.

^8^Local memory in OpenCL is the same thing as shared memory in CUDA.

^9^https://github.com/wanderine/NonSeparableFilteringCUDA

^10^https://github.com/wanderine/BROCCOLI/tree/master/code/testing_scripts

^11^http://www.fil.ion.ucl.ac.uk/spm/data/face_rep/face_rep_spm5_batch.m
